# Sound touch elastography of Achilles tendons in patients with type 2 diabetes mellitus versus healthy adults

**DOI:** 10.1186/s13098-023-01148-0

**Published:** 2023-08-21

**Authors:** Xinxin Huang, Xingyu Chen, Xiu Chen, Ping Chi, Pengfei Wang, Xiaomei Zhan, Chunpeng Zou, Liang Wang, Yanyan Dong

**Affiliations:** Department of Ultrasonic Diagnosis, The Second Affiliated Hospital and Yuying Children’s Hospital of Wenzhou Medical University, 1111 Wenzhou Avenue, Longwan District, Wenzhou, 325000 China

**Keywords:** Achilles tendon, Type 2 diabetes mellitus, Elasticity imaging technique, Sound touch elastography

## Abstract

**Background:**

The studies of the effect of diabetes on the stiffness of Achilles tendon (AT) tissue remain inconclusive, we believe it is necessary to find a reliable method which can be used to detect the stiffness changes of the AT in the diabetic state. The objective of the present study was to investigate the effectiveness of sound touch elastography (STE) as a tool for detecting diabetic Achilles tendinopathy.

**Methods:**

We conducted a retrospective review of 180 participants, consisting of 82 patients with type 2 diabetes mellitus (T2DM) and 98 healthy adults, who had undergone AT ultrasonography. Young ‘s modulus (E) values of the distal, middle, and proximal segments of bilateral ATs of all participants were measured using STE technique. The E values of each AT segment between the case and control group were compared.

**Results:**

The E values of the three segments of ATs in T2DM patients were lower than the healthy controls (*P* < 0.05). In both groups, the E values of the distal segments were lower than those of the middle segments, and the latter were lower than those of the proximal segments (*P* < 0.05). The E value of each segment of AT was inversely related to FPG, HbA1c, and diabetes duration (*P* < 0.05). The best cut-off points for the E values of the three segments of the AT for detecting diabetic tendinopathy were 347.44 kPa (AUC, 0.779), 441.57 kPa (AUC, 0.692), and 484.35 kPa (AUC, 0.676), respectively.

**Conclusion:**

STE can be used as a complementary diagnostic tool for the diagnosis of diabetic Achilles tendinopathy.

## Background

Type 2 diabetes mellitus (T2DM) is a complex chronic metabolic disease characterized by hyperglycemia, which is one of the most common chronic diseases in clinical practice. It can lead to complications involving various vital tissues and organs of the body [1, 2]. Studies have shown that T2DM not only leads to lower limb nerve and vascular lesions but also causes lower limb muscles, tendons, ligaments and cartilage lesions [3]. Persistent hyperglycemia has been found to induce a series of abnormal changes in the Achilles tendon (AT) and lead to tendinopathy during the development and progression of diabetes by some researchers [4, 5]. As is well known, diabetic foot is one of the most serious complications of T2DM and the main cause of disability in T2DM patients [6]. For decades, diabetic foot is thought to result mainly from neuropathy and ischemia. However, recent studies suggest that Achilles tendinopathy may precede neuropathy, which may be related to the development of diabetic foot [3, 7]. Therefore, early detection and treatment of Achilles tendinopathy are important for delaying its progression and reducing the risk of diabetic foot.

Currently, both Magnetic resonance imaging (MRI) and ultrasound are regarded as the gold standard for the diagnosis of AT disease [8, 9]. Although MRI has the advantages of radiation-free, high spatial resolution, excellent soft tissue contrast, and is capable of multiplanar and multi-sequence imaging, it is relatively expensive. And both traditional ultrasound and MRI lack of quantitative assessment of the biomechanical properties of the AT. Therefore, an imaging tool which enables early and accurately assessment of the biomechanical properties of AT tissue is urgently needed.

Sound touch elastography (STE) is a relatively new shear wave ultrasound elastography technique applied to quantitatively assessment of tissue stiffness by using acoustic radiation force to introduce shear waves in tissues. Currently, it has been widely used in the clinical detection of thyroid, breast and liver diseases [10–12]. Young’s modulus (E) is a measurement index of STE and is considered a reliable biomechanical index for reflecting soft tissue stiffness [11]. It changes associated with physiological and pathological conditions and has very important reference value for the diagnosis of diseases [13]. This study aims to estimate the E values of ATs in patients with T2DM as well as healthy adults by using the STE technique and investigate its potential clinical value.

## Materials and methods

### Subjects

The study was carried out according to the Helsinki Declaration (revised 2013) and was approved by our hospitals Medical Ethics Committee (approval number: 2022-K-333-01). Individual consent was waived due to the retrospective nature of this research.

We reviewed the medical records of subjects who had undergone AT ultrasonography in our hospital between October 2021 and January 2023. Subjects’ medical data were retrieved from the hospital electronic medical record system using the search term “AT stiffness”. T2DM patients who met the 1999 World Health Organization diagnostic criteria as well as healthy individuals were enrolled in the research. Inclusion criteria: age 45–75 years. Exclusion criteria: (I) prior history of foot trauma or foot surgery; (II) Achilles tendinitis caused by trauma or infection; (III) patients with rheumatic diseases, severe neurological disorder and other metabolic diseases such as abnormal obesity; (IV) athletes who engage in regular training or competition. We excluded 36 subjects with incomplete clinical data and 4 subjects who met the exclusion criteria. In total, 82 T2DM patients and 98 healthy subjects were included in this research. Patients with T2DM were assigned to the case group, healthy adults to the control group. Each subject ‘s clinical data, including sex, age, body mass index (BMI), diabetes-related complications, diabetes duration, treatment for diabetes, hemoglobin A1c (HbA1c), fasting plasma glucose (FPG), AT thickness, and the E value of AT were recorded.

### AT ultrasonography

AT ultrasonography was performed using Mindray Resona 7T ultrasound diagnostic instrument (Shenzhen Mindray Bio-Medical Electronics Co., Ltd, Shenzhen, China), equipped with a STE mode and a L11–3U linear array transducer (5.6–10.0 MHz). During the examination, the musculoskeletal program was selected.

**Fig. 1:**
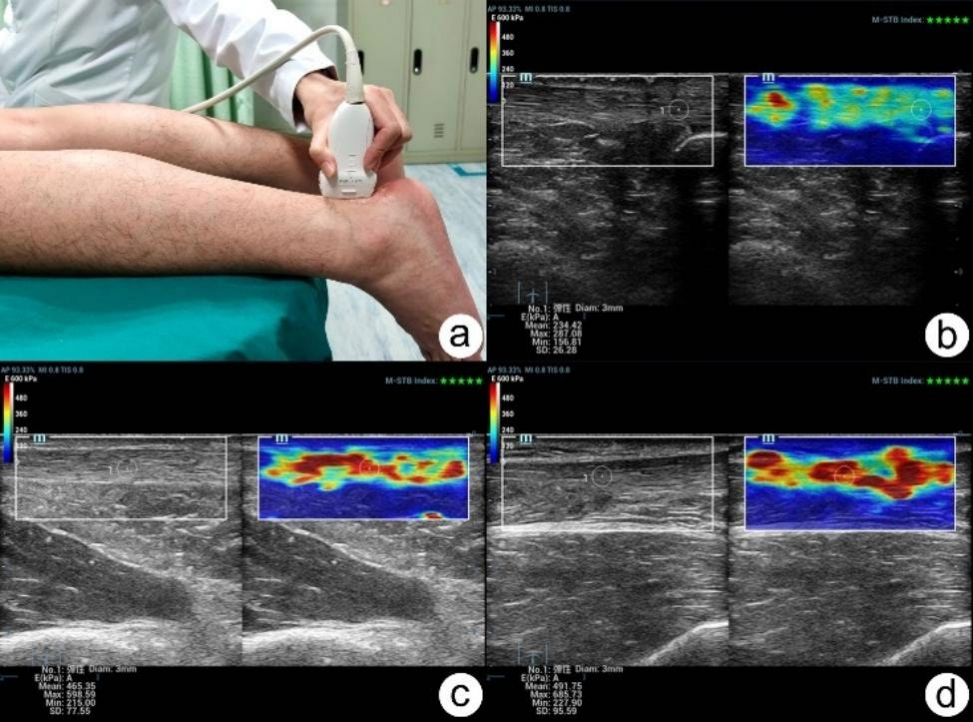
AT ultrasonography. (**a**) Subject position during the examination. Each subject was requested to lie on the examination bed in prone position, with both feet suspended over the edge of the bed fully relaxed. (**b**) STE examination of the distal segment of AT. The measured Emean value of the distal segment of AT was 234.42 kPa. (**c**) STE examination of the middle segment of AT. The measured Emean value of the middle segment of AT was 465.35 kPa. (**d**) STE examination of the proximal segment of AT. The measured Emean value of the proximal segment of AT was 491.75 kPa. AT, Achilles tendon; STE: Sound touch elastography; Emean: mean Young ‘s modulus

**Fig. 2:**
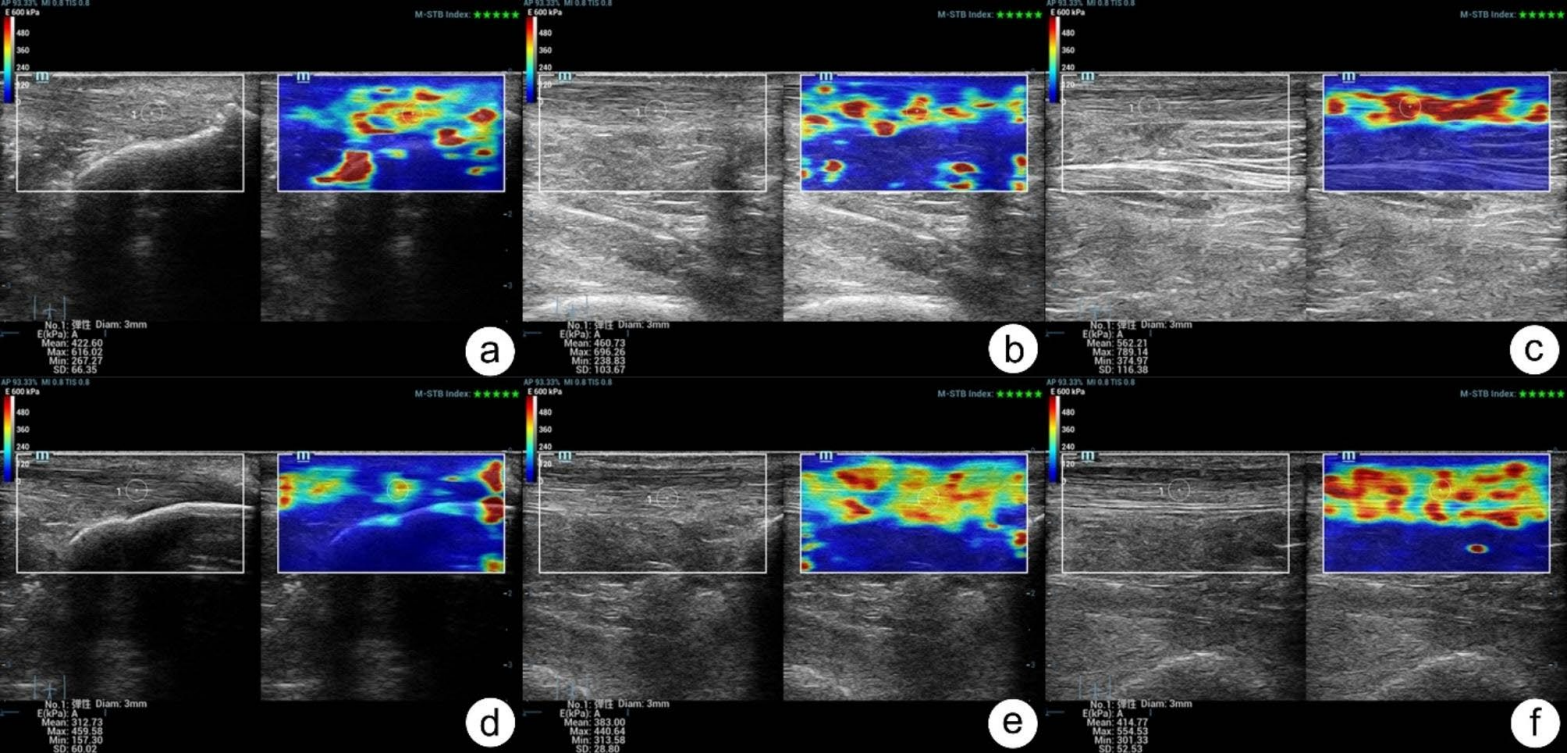
Images of AT of T2DM patients and healthy adults obtained by STE technology. (**a**) A 64-year-old healthy male. The measured Emean value of the distal segment of AT was 422.60 kPa. (**b**) A 64-year-old healthy male. The measured Emean value of the middle segment of AT was 460.73 kPa. (**c**) A 64-year-old healthy male. The measured Emean value of the proximal segment of AT was 562.21 kPa. (**d**) A 66-year-old male patient with T2DM. The measured Emean value of the distal segment of AT was 312.73 kPa. (**e**) A 66-year-old male patient with T2DM. The measured Emean value of the middle segment of AT was 383.00 kPa. (**f**) A 66-year-old male patient with T2DM. The measured Emean value of the proximal segment of AT was 414.77 kPa. AT, Achilles tendon; T2DM: type 2 diabetes mellitus; STE: Sound touch elastography; Emean: mean Young ‘s modulus

Each subject was requested to lie on the examination bed in prone position, with both feet suspended over the edge of the bed fully relaxed (Fig. 1a). The probe was placed on the surface of the AT and perpendicular to it, and thick coupling agent was filled between the probe and skin. To avoid any anisotropic artifacts, the probe was adjusted as parallel as possible to the long axis of the AT. Firstly, gray-scale ultrasonography was performed. On the transverse section of the AT, its maximum anteroposterior diameter was measured as its thickness. On the longitudinal section, its internal echo and fibrous structure was assessed. After the standard ultrasound examination, the STE mode was used. We chose Young ‘s modulus (E) as the stiffness metric. Meanwhile, the scale was adjusted to 28 and the range of E was adjusted to 0–600 kPa. At STE examination, the AT was divided into three segments: distal segment (the distal tendon-to-bone attachment zone), middle segment (2 to 6 cm above the tendon-bone junction), and proximal segment (the proximal musculo-tendinous junction) [4]. Each segment was detected separately. According to the motion stability (M-STB) index displayed on the top right corner of the screen, only when the M-STB index reached 4 and above, relatively stable stiffness data could be obtained. Thus, following image stabilization, a stiffness measurement was taken. The center of each AT segment of a 3 mm diameter was selected as the region of interest (ROI), avoiding calcified region (Figs. 1b–d and 2). Each segment of bilateral ATs was measured three times, and the mean was taken for statistical analysis. All subjects were examined by two independent sonographers.

### Statistical analysis

Firstly, variables were tested for normality of distribution using the Kolmogorov-Smirnov test. Continuous variables with normal distribution were presented as the mean ± standard deviation (SD). The intra-observer and inter-observer agreements were evaluated by the intraclass correlation coefficient (ICC). Next, the thickness and the E values of bilateral ATs were compared using independent-samples t-test. The E values of each AT segment between the case and control group were compared using t test, as well as the AT thickness. The comparison of E among the three segments of the AT in the control group was analyzed by one-way analysis of variance (ANOVA), and the LSD-t-test was used for pairwise comparisons. The comparison of E among the three segments of the AT in the T2DM group was analyzed by Kruskal-Wallis ANOVA test, followed by post hoc Bonferroni correction. Relationships between variables were tested using two-sided Pearson correlation analysis. Continuous values with non-normal distribution were presented as median (interquartile range). Comparison of two groups was analysed by a Mann-Whitney nonparametric test. Relationships between variables were tested using two-sided Spearman rank correlation. Receiver operator characteristics (ROC) curve and area under the curve (AUC) with a 95% confidence interval (CI) was used to estimate the validity of the AT E in the diagnosis of diabetic tendinopathy. Chi-square test was applied to compare categorical data. *P* value of less than 0.05 was considered for statistical significance. All analyses mentioned above were performed using SPSS (IBM, Chicago, IL, USA), version 26.0.

## Results

### General data of subjects

Subjects’ age, sex ratio, and BMI were not statistically different between the cases and controls (*P* > 0.05). Levels of FPG and HbA1c were significantly higher in cases as compared to controls (*P* < 0.05). The range of diabetes duration of T2DM patients was 4 years to 17 years, and the median time was 7 years. 10 patients with diabetic complications including atherosclerosis (10 cases), diabetic retinopathy (7 cases), diabetic nephropathy (4 cases) and diabetic neuropathy (3 cases), while 72 patients did not have diabetes-related complications. 7 patients did not receive any T2DM medication, 4 patients adopted diet adjustment and exercise by themselves, 66 patients had taken T2DM medication but with inadequate compliance, 5 patients took medication strictly adhered to medical orders (Table 1).

**Table 1:** Comparison of general data of the case and control group

Variable	Control Group (n = 98)	Case Group (n = 82)	*P* value
Age (years)	59.40 ± 6.44	60.10 ± 3.91	0.371
Sex (male/female)	60/38	45/37	0.390
BMI (kg/m^2^)	24.56 ± 1.71	24.76 ± 2.34	0.511
FPG (mmol/L)	5.20 (1.50)	9.88 (4.12)	< 0.001
HbA1c (%)	5.48 ± 0.19	8.66 ± 1.13	< 0.001
Diabetes duration (years)	0	7 (2)	/
Diabetes-related complications (with/without)	0/98	10/72	/
Treatment of diabetes (normalized/non-normalized)	0/0	5/77	/

Sex, diabetes duration and treatment of diabetes are presented as numbers. FPG is presented as median (interquartile range). Other data are expressed as mean ± SD. BMI, body mass index; FPG, fasting plasma glucose; HbA1c, hemoglobin A1c

### Intra-observer and inter-observer agreement

The intra-ICC for the three times E measurements of the distal, middle, and proximal segments of the AT for the sonographer A were 0.823, 0.828, and 0.817, respectively, and for the sonographer B were 0.810, 0.771, and 0.805, respectively. The inter-ICC for the E measurements of three segments of the AT by two independent sonographers were 0.787, 0.810, and 0.826, respectively (Table 2).

**Table 2:** Intra-observer and Inter-observer agreement

AT	Intra-ICC (n = 360)		Inter-ICC (n = 360)
	Sonographer A	Sonographer B	
Distal segment	0.823	0.810	0.787
Middle segment	0.828	0.771	0.810
Proximal segment	0.817	0.805	0.826

n, Achilles tendon number; AT, Achilles tendon; ICC, intraclass correlation coefficient

### Comparison of bilateral ATs

There was no statistically significant difference in the thickness of bilateral ATs within the case and control group (*P* > 0.05). No statistical difference was observed in the E values of the three segments of bilateral ATs within the case and control group (*P* > 0.05) (Table 3).

**Table 3:** Comparison of bilateral ATs in the case and control group

Variable	Control Group (n = 98)				Case Group (n = 82)		
	Left	Right	*P* value		Left	Right	*P* value
AT number	98	98	*/*		82	82	*/*
AT Thickness (mm)	5.19 ± 0.53	5.21 ± 0.53	0.758		5.31 ± 0.56	5.30 ± 0.55	0.900
E of AT (kPa)							
Distal segment	370.37 ± 57.53	376.41 ± 49.59	0.431		320.53 ± 38.92	330.32 ± 39.04	0.110
Middle segment	452.26 ± 51.00	452.95 ± 42.20	0.918		423.06 ± 54.10	413.88 ± 49.20	0.257
Proximal segment	482.88 ± 49.15	494.11 ± 57.62	0.144		456.38 ± 58.00	452.44 ± 53.32	0.652

AT number is presented as numbers. Other data are expressed as mean ± SD. AT, Achilles tendon; E, Young’s modulus

### Comparison of ATs between groups

The E values of the three segments of the AT in the case group were significantly lower than those of the control group (325.43 ± 39.17 vs. 373.39 ± 53.65 kPa; *t* = − 9.782; *P* < 0.05) (418.47 ± 51.76 vs. 452.61 ± 46.69 kPa; *t* = − 6.575; *P* < 0.05) (454.41 ± 55.57 vs. 488.49 ± 53.71 kPa; *t* = − 5.902; *P* < 0.05), respectively. Comparison of the AT thickness between the case and control group was not statistically significant (5.31 ± 0.56 vs. 5.20 ± 0.53 mm; *t* = 1.835; *P* = 0.067). Within the case and control group, the E values were lower in the distal segments than in the middle and proximal segments (*P* < 0.05). Then the E values were lower in the middle segments than in the proximal segments (*P* < 0.05) (Table 4).

**Table 4:** Comparison of the thickness and the E values of the ATs between the case and control group

Variable	Control Group (n = 98)	Case Group (n = 82)	*P* value
AT number	196	164	/
AT Thickness (mm)	5.20 ± 0.53	5.31 ± 0.56	0.067
E of AT (kPa)			
Distal segment	373.39 ± 53.65^a, b^	325.43 ± 39.17^a, b^	< 0.001
Middle segment	452.61 ± 46.69^c^	418.47 ± 51.76^c^	< 0.001
Proximal segment	488.49 ± 53.71	454.41 ± 55.57	< 0.001
*P* value	< 0.001	< 0.001	

AT number is presented as numbers. Other data are expressed as mean ± SD. ^a^: *P* < 0.001 (Distal segment vs. Middle segment); ^b^: *P* < 0.001 (Distal segment vs. Proximal segment); ^c^: *P* < 0.001 (Middle segment vs. Proximal segment). AT, Achilles tendon; E, Young’s modulus

### Correlation analysis for the E of AT

**Fig. 3:**
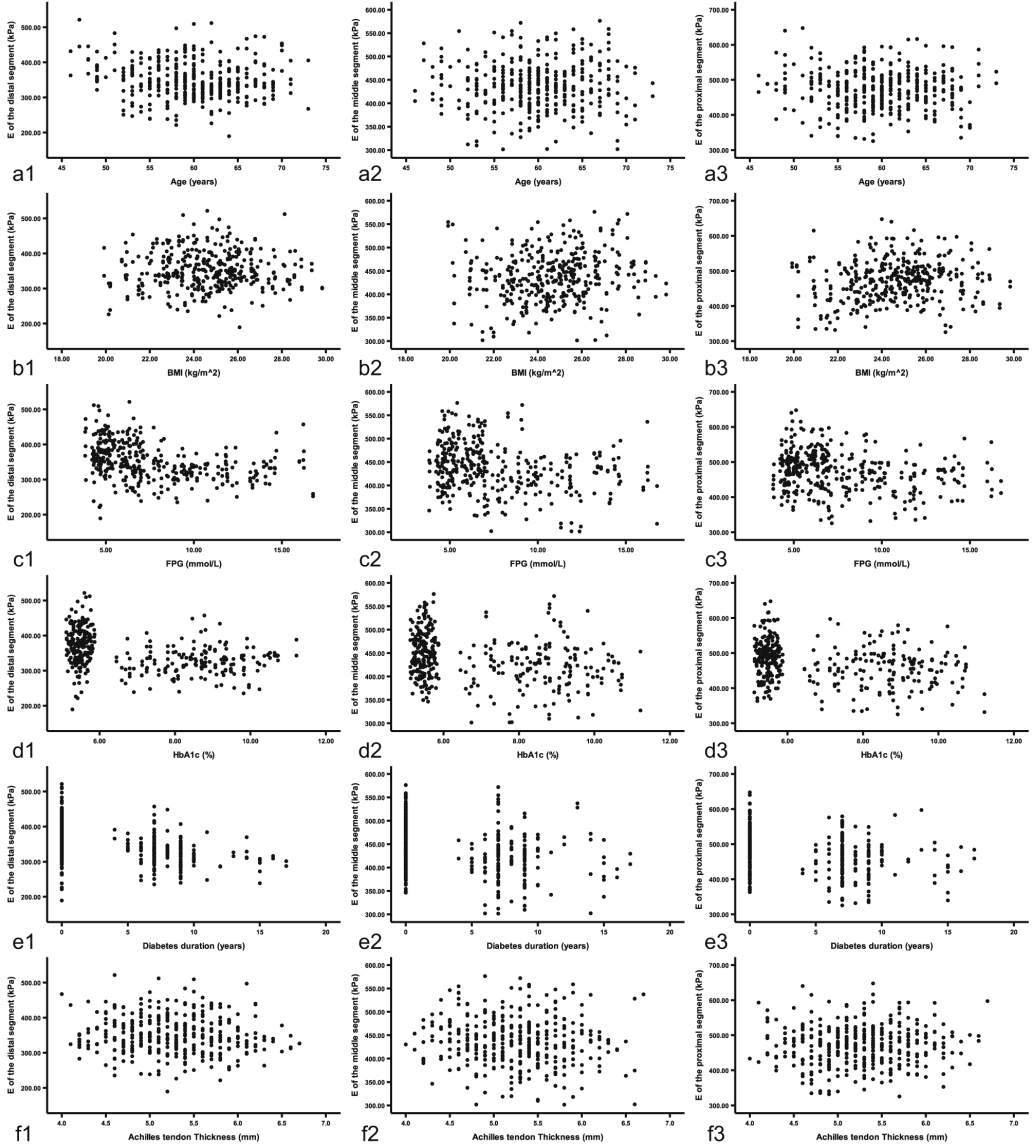
Scatterplots of relationships of the E of each segment of AT and clinical or biochemical parameters. (a1–3) The E value of the distal segment was inversely related to age (*r* = − 0.122; *P* < 0.05). The E values of the middle and proximal segments were not related to age (*P* > 0.05). (b1 − 3) The E value of the distal segment was not related to BMI (*P* > 0.05). The E values of the middle and proximal segments were positively related to BMI respectively (*r* = 0.109; *r* = 0.142; *P* < 0.05). (c1 − 3) The E values of the three segments of the AT were inversely related to FPG respectively (*r* = − 0.424; *r* = − 0.274; *r* = − 0.229; *P* < 0.05). (d1 − 3) The E values of the three segments of the AT were inversely related to HbA1c respectively (*r* = − 0.376; *r* = − 0.289; *r* = − 0.260; *P* < 0.05). (e1 − 3) The E values of the three segments of the AT were inversely related to diabetes duration respectively (*r* = − 0.507; *r* = − 0.313; *r* = − 0.296; *p* < 0.05). (f1–3) The E value of each segment was not related to AT thickness (P > 0.05). E, Young’s modulus; AT, Achilles tendon; BMI, body mass index; FPG, fasting plasma glucose; HbA1c, hemoglobin A1c

The E value of each segment of the AT was inversely correlated with FPG, HbA1c and diabetes duration respectively (*r*_*distal*_ = − 0.424; *r*_*distal*_ = − 0.376; *r*_*distal*_ = − 0.507; *P* < 0.05) (*r*_*middle*_ = − 0.274; *r*_*middle*_ = − 0.289; *r*_*middle*_ = − 0.313; *P* < 0.05) (*r*_*proximal*_ = − 0.229; *r*_*proximal*_ = − 0.260; *r*_*proximal*_ = − 0.296; *P* < 0.05). The E value of distal segment was inversely related to age (*r* = − 0.122; *P* < 0.05). Additionally, the E values of the middle and proximal segments were positively related to BMI respectively (middle, *r* = 0.109; *P* < 0.05) (proximal, *r* = 0.142; *P* < 0.05) (Fig. 3).

### ROC curve analysis

**Fig. 4:**
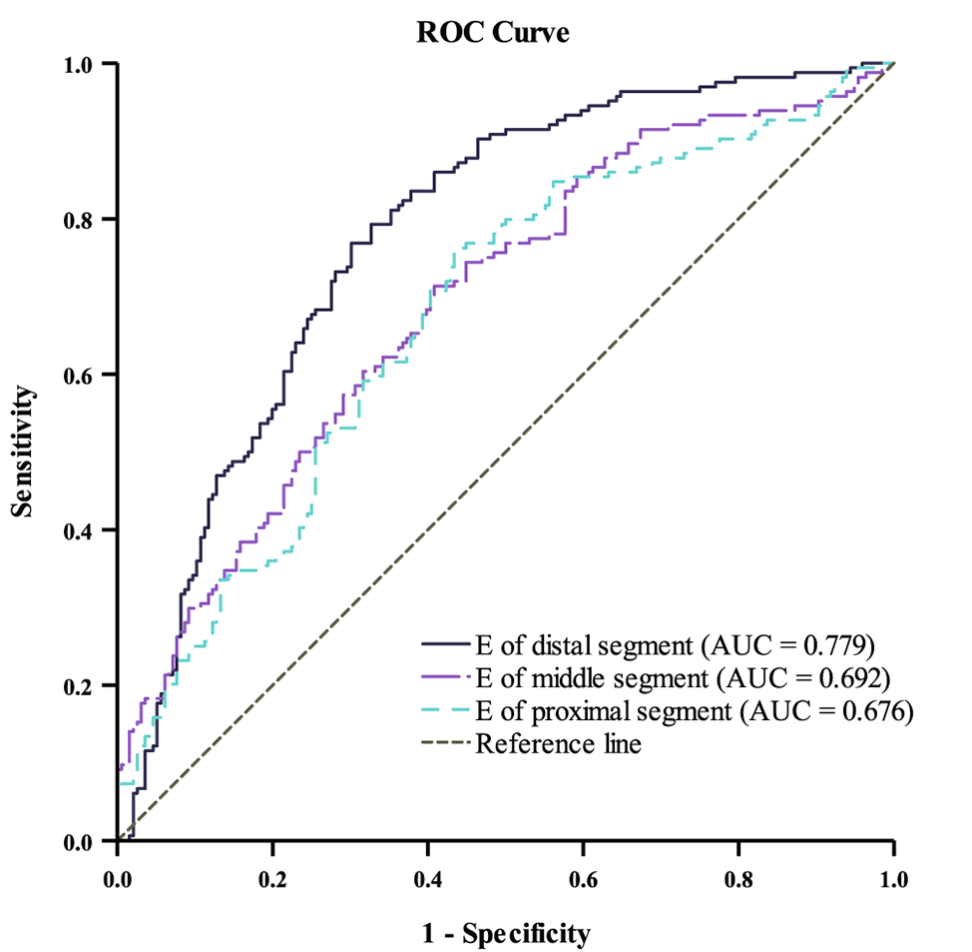
The ROC curve of AT E for detecting diabetic tendinopathy with the sensitivity and the 1 − specificity as the ordinate and abscissa respectively. The optimal cutoff point for the E of the distal segment of AT was 347.44 kPa (sensitivity, 76.8%; specificity, 69.9%; AUC, 0.779; 95% CI, 0.731–0.827), for the middle segment was 441.57 kPa (sensitivity, 71.3%; specificity, 59.2%; AUC, 0.692; 95% CI, 0.638–0.746), and for the proximal segment was 484.35 kPa (sensitivity, 75.6%; specificity, 56.6%; AUC, 0.676; 95% CI, 0.621–0.732). ROC, receiver operator characteristics; E, Young’s modulus; AUC, area under the curve; CI, confidence interval

Regarding the validity of the E as a predictor of diabetic tendinopathy of the AT, ROC analysis showed that the optimal cutoff value for the E of the distal segment was 347.44 kPa (sensitivity, 76.8%; specificity, 69.9%; AUC, 0.779; 95% CI, 0.731–0.827); for the middle segment, it was 441.57 kPa (sensitivity, 71.3%; specificity, 59.2%; AUC, 0.692; 95% CI, 0.638–0.746); and for the proximal segment, it was 484.35 kPa (sensitivity, 75.6%; specificity, 56.6%; AUC, 0.676; 95% CI, 0.621–0.732) (Fig. 4).

## Discussion

The AT is the largest, thickest, and strongest tendon in the human body, which together with the plantar fascia play essential role in foot biomechanics [14]. Previous studies have shown that long-term hyperglycemic conditions can lead to excessive accumulation of advanced glycosylation endproducts (AGEs) in the AT, resulting in the destruction of collagen fibers in the AT tissue [15, 16]. Changes in the tissue structure of the AT adversely affect foot biomechanics and can lead to abnormal alterations in gait and foot loading patterns, which may be one of the causes of diabetic foot ulcer attacks in diabetic patients [3, 4, 17]. Therefore, early detection of Achilles tendinopathy in T2DM patients may play a role in preventing the occurrence of diabetic foot ulcers.

Current methods used for assessing AT stiffness are still under investigation. Several previous studies have reported that real-time sonoelastography can be used to assess AT stiffness [4, 18]. However, this technique is highly dependent on the operator’s experience and does not quantitatively measure AT stiffness. In recent years, a non-invasive muscle elasticity tester called “MyotonPRO” has been used to assess AT stiffness [19, 20]. But this technique lacks imaging information and cannot accurately measure a certain point. Therefore, we need a method that can clearly image and quantitatively measure AT stiffness, and STE meets these advantages. In this study, the tissue stiffness of each segment of bilateral ATs in T2DM patients and healthy adults was assessed and compared to clarify whether STE can be used to differentiate ATs in the diabetic state from normal ATs.

In our research, the inter-observer and intra-observer reliability of E values measured by two different sonographers for each segment of the AT was good, with ICCs all greater than 0.75. This suggests that the STE technology exhibits excellent stability across various testers. This convenience makes STE applicable to multicenter studies and promotes its general application in clinical practice.

The present study demonstrated that in contrast to the controls, T2DM patients had significantly lower E values in all three segments of the AT. The lower E values of the AT, the lower the stiffness. This finding suggests that the ATs are softer in T2DM patients than the normal ATs. However, the effect of diabetes on the stiffness of AT tissue remains inconclusive [21–25]. Evranos et al. [4] assessed the AT stiffness in diabetic patients using real-time strain sonoelastography and found diabetic patients with foot ulcers had significantly lower stiffness in the middle and distal segments compared to the controls. Harish et al. [26] and Iyidir et al. [27] achieved the same conclusion using shear wave elastography and acoustic radiation force impulse elastography technique, respectively. It has been suggested that diabetes leads to changes in the tissue structure of the AT, which may be the result of excessive accumulation of AGEs [15, 16]. Changes in AT structure may cause changes in biomechanical properties, which may be a cause of softer in AT stiffness. Previous studies have revealed twisted and crossed collagen fibers in the longitudinal section of diabetic AT under electron microscopy [17]. This structural change of collagen fibers may increase anisotropic interference in the elastography of ATs, resulting in lower measured AT stiffness values. Therefore, we believe that STE technique allows for the precise assessment of tendon stiffness, which could in part reflect the pathological state of the AT in T2DM patients. The contrary conclusions drawn in other studies may be attributed to the differences in diabetes duration and glycemic control among the participants.

This study observed a trend towards thicker ATs in cases compared to healthy adults, but the difference was not statistically significant. This finding concurs with that of Giacomozzi et al. [3]. Investigators considered that pathological tendons could compensate for the disordered areas by increasing in thickness [28]. The lack of a statistically significant difference in AT thickness between cases and healthy adults in this study may be related to the inclusion of patients with shorter diabetes duration and fewer comorbidities. In addition, we found that E values of the AT showed a gradual decreasing trend from proximal to distal segments in both T2DM patients and healthy controls. This result aligns with Zhang et al. [29] and Helfenstein-Didier et al. [30], and may be related to the anatomical differences among different segments of the AT, such as the differential distribution of collagen fibers and different physiological functions.

Moreover, we found that the E value of each segment of the AT was negatively correlated with FPG, HbA1c, and diabetes duration, respectively. This indicates that AT stiffness may be related to the diabetes duration and glycemic control in T2DM patients, the longer the disease course and the poorer the glycemic control, the softer the AT. Previous researches have shown that BMI and age are risk factors for AT injury [31, 32]. However, our study only found that age was negatively correlated with E value of distal segment, and BMI was positively correlated with E values of the middle and proximal segments. This might also be associated with the differences in anatomical structure and function between segments of the AT. Indeed, there are many factors or variables affecting AT stiffness. Previous studies have shown that AT stiffness decreases with age [33]. There was no statistically significant difference in age between the two groups after we divided the patients into case and control groups, so this study could not analyze the effect of age on AT stiffness. In addition, it has been reported that frequent-exercisers had significantly higher E values of the AT, which may be related to the increased number and tight arrangement of collagen fibers in long-term exercisers [34]. In order to avoid the influence of exercise volume on the data, professional athletes were excluded at the time of the study. Furthermore, some other diseases (such as rheumatic diseases, uremia, familial hypercholesterolemia, etc.) that may affect AT stiffness were not included in this study [35–37].

Our findings revealed the optimal cutoff points for the E values of the three segments of the AT for detecting diabetic tendinopathy were 347.44 kPa (AUC, 0.779), 441.57 kPa (AUC, 0.692), and 484.35 kPa (AUC, 0.676), respectively. Generally, an AUC of over 0.9 indicates high diagnostic accuracy, 0.7 to 0.9 indicates medium accuracy, and 0.5 to 0.7 indicates low accuracy. Therefore, compared to the proximal and middle segments, the distal segment has the best diagnostic accuracy with 76.8% sensitivity and 69.9% specificity. Based on this, we recommend the distal segment as the optimal site of examination when using STE technique to evaluate the ATs in T2DM patients.

At present, elastography studies on the AT in T2DM patients are in a preliminary stage. Our study found that T2DM patients have lower AT stiffness compared to healthy adults, which may be a potential factor in the development of diabetic foot [4, 7]. Quantitative measurement of AT stiffness by STE may be a potential diagnostic method to predict the occurrence of diabetic foot, and early monitoring of AT stiffness changes in diabetic patients can effectively assist clinicians in better diagnostic analysis and scientific follow-up. This may help them intervene in patients in a timely manner (e.g., customized shoes or orthoses). As far as the current findings are concerned, we need to do further studies to explore the potential link between Achilles tendinopathy and diabetic foot in diabetic patients.

Certainly, there were some limitations in this research. Firstly, the patients enrolled in this research received different diabetic treatment regimens, which might affect the results. However, as this retrospective study did not provide standardized diabetes treatment, we did not subdivide patients by treatment regimen. Secondly, we only used STE technique to assess the stiffness of AT and did not combine other detection methods. Thirdly, we did not perform a quantitative assessment of physical activity levels in all subjects, such as activity time, type of activity item, and so on. Finally, we did not have pathological results for comparison.

## Conclusion

In conclusion, the STE technique can be used to distinguish diabetic tendinopathy from healthy AT. We prefer to use it as a complementary diagnostic tool for the diagnosis of diabetic Achilles tendinopathy. Furthermore, we recommend the distal segment of the AT as the optimal examination region.

## Data Availability

The data that support the findings of this study are available from the corresponding author upon reasonable request.
